# Mechanisms Underlying Myopia Progression from Visual Signaling to Metabolic Remodeling in Retina

**DOI:** 10.31662/jmaj.2025-0268

**Published:** 2025-08-01

**Authors:** Yajing Yang, Deokho Lee, Kate Gettinger, Kazuo Tsubota, Kazuno Negishi, Toshihide Kurihara, Yohei Tomita

**Affiliations:** 1 Department of Ophthalmology, Keio University School of Medicine, Tokyo, Japan; 2 Laboratory of Photobiology, Keio University School of Medicine, Tokyo, Japan; 3 Laboratory of Chorioretinal Biology, Keio University School of Medicine, Tokyo, Japan; 4 Tsubota Laboratory, Inc., Tokyo, Japan

**Keywords:** myopia progression, retinal signaling, ON/OFF pathways, rod pathway, retinal dysfunction, metabolic remodeling

## Abstract

Myopia has emerged as a major global health concern due to its rising prevalence and the associated risk of irreversible visual impairment. Although scleral and choroidal changes have traditionally received emphasis, recent studies highlight the retina as a key contributor to the onset and progression of myopia. This review incorporates recent advances across three interrelated domains: visual pathway modulation, functional impairment, and metabolic remodeling. Abnormal ON pathway signaling—particularly under mesopic lighting—disrupts emmetropization feedback and promotes ocular elongation. As myopia progresses, retinal dysfunction unfolds in a temporal cascade, beginning in the inner retina and eventually leading to widespread structural and functional degeneration. Concurrently, the retina exhibits stage-dependent metabolic shifts, progressing from early mitochondrial and lipid metabolism changes to mid-phase destabilization of membrane integrity, and culminating in late-stage oxidative stress, inflammation, and metabolic remodeling. Collectively, these findings redefine myopia as a progressive retinal disorder and underscore the potential of retina-targeted interventions to preserve homeostasis and mitigate long-term degeneration.

## Introduction

Myopia has emerged as one of the most pressing global public health challenges, with its prevalence reaching 35.81% according to the latest epidemiological data ^(1)^. In addition to its high prevalence, a subset of individuals develops pathological myopia ^(2)^, and the risk of associated complications increases significantly with high myopia. Specifically, the risk of myopic macular degeneration (MMD) has been reported to be 845 times higher, and the risk of retinal detachment (RD) 12.62 times greater—both of which can result in irreversible vision loss ^(3)^. These alarming trends have raised considerable concern among the general population ^(4)^. Therefore, investigating the mechanisms underlying the onset and progression of myopia is imperative.

Extensive studies over the past decades have provided substantial insight into axial elongation, particularly involving scleral remodeling and choroidal perfusion deficits. Recent findings suggest that endoplasmic reticulum (ER) stress, through the Protein Kinase R-like ER Kinase and Activating Transcription Factor 6 pathways ^(5)^, contributes to extracellular matrix remodeling, highlighting a link between intracellular stress and ocular structural changes. Concurrently, consistent findings of choroidal thinning and reduced blood flow in myopic eyes suggest that impaired choroidal perfusion may exacerbate scleral hypoxia and promote remodeling, ultimately driving axial elongation ^(6), (7), (8)^.

Despite extensive investigation of scleral remodeling and choroidal perfusion deficits with axial elongation, these mechanisms alone cannot fully explain the most vision-threatening complications of myopia. Major conditions such as MMD, retinoschisis, RD, and choroidal neovascularization predominantly affect the retina, emphasizing its central role in irreversible visual impairment ^(9)^. Beyond serving as a passive target of mechanical stress, emerging evidence suggests that the retina actively contributes to ocular growth regulation through alterations in visual signaling pathways, functional dynamics, and metabolic remodeling. Therefore, this review focuses on the retina’s involvement throughout the course of myopia—from early functional and molecular changes to progressive dysfunction—offering new insights into disease mechanisms and identifying potential targets for clinical intervention.

## Light-dependent Modulation of ON/OFF Pathways and Rod Involvement in Myopia Development

The retina consists of a series of well-organized neuronal layers interconnected by synapses, primarily comprising rod and cone photoreceptors, horizontal cells, bipolar cells, amacrine cells, and retinal ganglion cells (RGCs). Visual information is encoded via the ON and OFF pathways, which respond to luminance increments and decrements, respectively. These pathways are mediated by distinct populations of ON- and OFF-type bipolar and ganglion cells, each specialized in detecting changes in light intensity. Their activity is shaped not only by intrinsic retinal circuitry but also by the external visual environment ^(10), (11), (12), (13)^. Recent evidence suggests that alterations in ON or OFF pathway signaling may be associated with changes in ocular growth ^(14), (15)^. This section reviews animal and human studies that examine ON/OFF pathway alterations and rod-driven signaling in the regulation of ocular growth.

Animal studies have offered important insights into how ON pathway activity responds to myopia-related stimuli. Pan et al. ^(16)^ recorded RGC responses in mice and observed that ON RGCs exhibited reduced spiking activity and increased spontaneous firing under hyperopic defocus. Genetic studies have identified several key genes involved in ON pathway signaling. Loss-of-function mutations in genes such as *Nyx* and *Lrit3* result in abnormal retinal signaling and increased susceptibility to experimental myopia in mice ^(17), (18)^. In human studies, Westall et al. ^(19)^ reported that full-field electroretinography (ERG) b-wave amplitudes were reduced in myopic individuals, particularly under high-luminance conditions that predominantly activate cone-driven ON bipolar cells. However, not all reductions in ON pathway activity promote myopia. For instance, pharmacological blockade of ON bipolar cells with selective agents has been shown to slow form-deprivation myopia progression in chicks ^(20)^. These findings suggest that ON pathway dysfunction may influence ocular growth through distinct mechanisms. Persistent disruptions, such as genetic mutations, impair normal emmetropization and predispose the eye to axial elongation. In contrast, acute pharmacological suppression may transiently disrupt growth-promoting cascades and slow myopia progression. Further studies are required to clarify the temporal and functional roles of ON signaling in ocular growth regulation.

Ambient lighting conditions also influence retinal signaling and ON pathway activity. Under low-light or mesopic conditions, rod photoreceptors dominate input to the ON pathway via rod bipolar cells and AII amacrine cells, while cone-driven signals are reduced ^(21)^. Such conditions are common in modern environments. Prolonged exposure to mesopic lighting during daytime may sustain rod-driven ON signaling, disrupting the visual feedback necessary for emmetropization. Additionally, inadequate light exposure reduces retinal dopamine release, creating a signaling environment that favors axial elongation ^(22), (23), (24), (25)^.

However, not all low-light conditions promote myopia. Studies show that scotopic environments—such as starlight or near-complete darkness—do not stimulate axial elongation in animals or children ^(26)^. One explanation is that these environments lack sufficient spatial and temporal contrast to generate effective retinal defocus cues. Alternatively, the absence of meaningful visual input may suppress activation of the emmetropization pathway entirely. In such scenarios, retinal circuits involved in growth regulation may remain inactive, resulting in a form of visual “silence” that neither promotes nor inhibits eye growth. These findings underscore the importance of both light intensity and visual input quality in ocular development.

Beyond light intensity, the spatial characteristics of visual stimuli—particularly contrast—also critically modulate retinal pathway activity. High-contrast patterns preferentially activate the OFF pathway, which responds to luminance decrements and spatial edges. In contrast, the ON pathway is more responsive to luminance increments and is preferentially activated by low-contrast or diffuse stimuli ^(27), (28)^. Excessive OFF pathway activation, especially under prolonged high-contrast exposure, has been implicated in promoting axial elongation. Accordingly, contrast reduction strategies—such as minimizing sharp edges in the visual field—have been proposed as potential interventions for myopia control ^(29)^. By attenuating OFF-dominant signaling while preserving ON pathway input, such strategies may help restore a functional balance in retinal processing to support emmetropization.

Despite evidence that excessive OFF pathway activation may promote axial elongation, the structural integrity of this pathway does not appear essential for normal refractive development. For example, knockout of Vsx1, which impairs OFF bipolar cell development, does not affect emmetropization in mice ^(30)^.

In contrast, alterations in ON pathway activity are consistently observed in myopic eyes, although the causal relationship with axial elongation remains unclear. ON dysfunction may contribute to abnormal ocular growth or represent secondary remodeling from sustained axial elongation. Additional studies are needed to clarify the sequence and mechanisms of ON pathway changes during myopia development.

**Figure 1.**
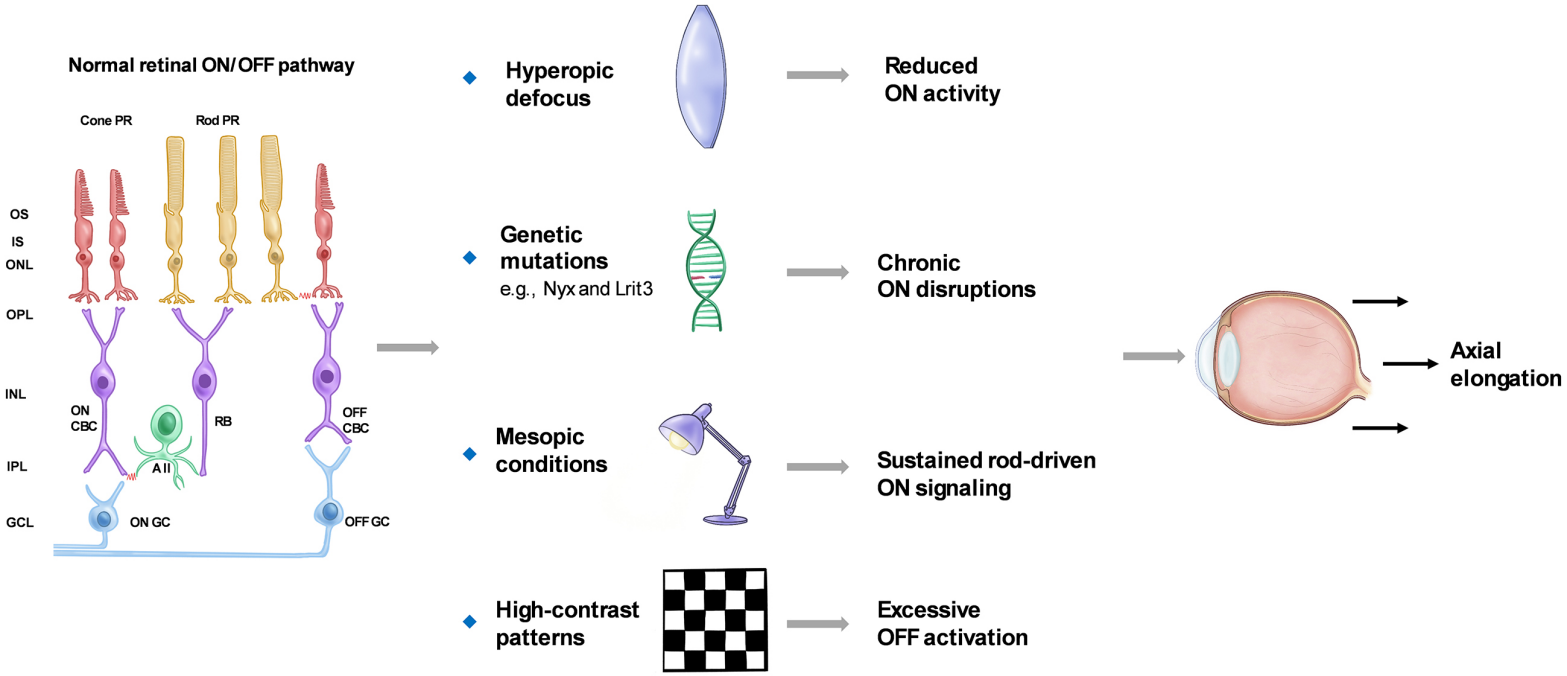
Effects of visual conditions and ON/OFF pathway alterations on axial elongation. This schematic illustrates how visual and genetic factors disrupt ON/OFF signaling and promote axial elongation. Hyperopic defocus impairs ON pathway signaling and disrupts feedback control of axial growth. Nyx and Lrit3 mutations impair ON signaling and increase myopia susceptibility. Mesopic light sustains rod-driven ON input, while high contrast enhances OFF activation. These changes disrupt emmetropization feedback and drive ocular elongation. PR: Photoreceptor; OS: outer segment; IS: inner segment; ONL: outer nuclear layer; OPL: outer plexiform layer; INL: inner nuclear layer; IPL: inner plexiform layer; GCL: ganglion cell layer; CBC: cone bipolar cell; RB: rod bipolar cell.

Together, these findings highlight how light-dependent modulation of ON/OFF pathways and rod-driven signaling contributes to the regulation of ocular growth. Figure 1 provides a schematic summary of these pathway interactions and their involvement in myopia development.

Beyond pathway-specific alterations, cumulative functional impairments—including neural stress, compensatory adaptation, and eventual synaptic disruption—emerge alongside structural remodeling. The following section reviews electrophysiological and anatomical evidence illustrating how myopia progressively impairs overall retinal function.

## Progressive Retinal Dysfunction in Long-term Myopia

Progressive myopia is increasingly recognized not only as a condition involving ocular elongation but also as one associated with time-dependent retinal dysfunction ^(31)^. Beyond the mechanical consequences of axial elongation, sustained myopic stress may induce cumulative neural strain, synaptic dysregulation, and gradual disruption of retinal signaling. Notably, functional deterioration often emerges subtly and progresses over time, even when gross structural changes appear stable ^(32)^. These evolving functional deficits can impair visual processing efficiency and reflect deeper dysfunction within retinal circuitry.

ERG is widely used to assess retinal function, with distinct waveform components corresponding to the activity of specific neuronal populations ^(33)^. The a-wave primarily represents photoreceptor activity and serves as an indicator of outer retinal integrity. The b-wave and oscillatory potentials (OPs), generated predominantly by bipolar and amacrine cells, respectively, reflect the functional status of the inner retina.

In children and adolescents, retinal function typically remains within normal limits among myopic individuals, except in cases of high myopia ^(34), (35)^. However, as myopia persists into adulthood, early functional stress responses may emerge. For instance, Chen et al. ^(36)^ reported significantly shortened implicit times of OPs on multifocal ERG in young adults with progressive myopia, while amplitudes remained unchanged. Similarly, Wan et al. ^(37)^ observed altered peak frequencies of rod-driven OPs in myopic young adults compared to emmetropic controls. This pattern—reduced implicit time without amplitude loss—may reflect an early adaptive response of the retina to sustained physiological stress. It has been proposed that prolonged stress modulates response timing as a compensatory mechanism, enabling the retina to maintain output efficiency under continued demand ^(38)^. A similar process may occur in early myopia progression, where faster response timing helps preserve visual signaling despite increasing neural strain.

As myopia progresses, both OP and b-wave amplitudes begin to decline, accompanied by delayed implicit times ^(39)^, indicating advancing dysfunction of the inner retinal circuitry. Although compensatory mechanisms may initially preserve synaptic transmission, they appear insufficient to sustain retinal efficiency as the condition worsens. Concurrently, structural alterations become more apparent, with progressive thinning observed in the inner retinal layers ^(40)^, suggesting that functional and morphological degeneration occur in parallel with continued axial elongation.

In long-standing high myopia, retinal dysfunction may extend beyond the inner retina to affect photoreceptors. Under these conditions, reduced a-wave amplitudes have been reported ^(41), (42)^, indicating compromised outer retinal function. Epidemiological studies further support that the risk of myopia-related complications increases not only with greater axial length but also with longer myopia duration ^(43)^, reinforcing the concept of a time-dependent continuum involving retinal stress, functional decline, and structural degeneration.

Thus, myopia-related retinal dysfunction may be conceptualized as a temporal cascade—beginning with early compensatory adaptions, progressing to functional decline, and culminating in structural degeneration. Recognizing this trajectory is essential not only for understanding the pathophysiological mechanisms of myopia but also for identifying early intervention windows to prevent irreversible vision loss. Future research should prioritize the detection of early biomarkers of retinal stress, enhance sensitivity to subclinical functional changes, and develop neuroprotective strategies aimed at interrupting or slowing this degenerative process.

## Metabolic Remodeling and Molecular Alterations in Myopic Retinas

In addition to electrophysiological and structural remodeling, myopia triggers a dynamic, stage-dependent cascade of molecular and metabolic changes in the retina. Initially, these alterations may serve as adaptive responses to local stress but can progressively lead to pathological remodeling as myopia advances.

In the early phase of myopia development, the retina initiates a coordinated metabolic response involving multiple pathways. Transcriptomic analyses in the form-deprivation myopia (FDM) chick model have shown early upregulation of mitochondrial energy metabolism pathways—particularly the tricarboxylic acid cycle and oxidative phosphorylation—within 72 hours after form deprivation ^(44)^. Similarly, in the lens-induced myopia (LIM) model in guinea pigs, early activation of lipid metabolism has been observed, as evidenced by increased levels of ceramide-related enzymes and differential expression of proteins involved in sphingolipid biosynthesis within four days of induction ^(45)^. These findings suggest that the retina undergoes metabolic reprogramming before any overt structural or functional deterioration becomes apparent. Such early responses likely represent adaptive mechanisms aimed at preserving neuronal function and maintaining retinal homeostasis under biomechanical and metabolic stress.

As myopia progresses, early lipid-related responses give way to a more persistent disruption of membrane lipid homeostasis. In the guinea pig FDM model, significant downregulation of membrane-associated fatty acids—including arachidic acid, cholesterol, and octadecanoic acid—has been reported after two weeks of deprivation ^(46)^. Likewise, in the guinea pig LIM model, retinal expression of bone morphogenetic protein 2, a key regulator of lipid metabolism and tissue remodeling, is markedly reduced after three weeks ^(47)^. These mid-phase alterations suggest that sustained axial elongation undermines the retina’s ability to maintain lipid composition and membrane integrity, marking a transition from metabolic adaptation to structural vulnerability.

Beyond lipid remodeling, synaptic signaling pathways are also disrupted during the intermediate stage of myopia development. Studies in the mouse FDM model have demonstrated significant activation of the Wnt2b/Fzd5/β-catenin signaling pathway in the retina after four weeks of visual deprivation ^(48)^. Given the well-established role of canonical Wnt signaling in synaptic organization and neuroplasticity ^(49)^, its upregulation in myopic retinas suggests that prolonged defocus may impair retinal synaptic transmission, potentially affecting downstream signaling cascades involved in ocular growth regulation.

As myopia becomes chronic and progresses to high myopia, metabolic remodeling shifts from adaptive to pathological. In the guinea pig FDM model, the accumulation of reactive oxygen species and lipid peroxidation products reflects a breakdown in antioxidant defenses, resulting in oxidative damage to retinal neurons and supporting glial cells ^(50)^. In humans with long-standing high myopia, Luminex-based analysis of aqueous humor samples has shown elevated levels of pro-inflammatory cytokines, including interleukin-6 and intercellular adhesion molecule-1, indicating a persistent low-grade inflammatory state ^(51)^.

Retinal glial cells may act synergistically to promote localized inflammatory responses ^(52)^. Among these, Müller cells are particularly sensitive to hypoxia due to their high expression of hypoxia-inducible factor 1 (HIF-1) ^(53)^. In response to hypoxic stress, they initiate vascular endothelial growth factor (VEGF) signaling and contribute to retinal metabolic remodeling. The inflammatory signals they release may also activate microglia, which subsequently secrete chemokines such as CCL2, promoting peripheral immune cell infiltration and compromising the retinal barrier ^(54)^.

Metabolic disruption is often accompanied by vascular and structural remodeling. In the FDM mouse model, aberrant activation of HIF-1α and VEGF signaling pathways contributes to choroidal neovascularization and reduced retinal microcirculation ^(55)^. In a more advanced model of pathological myopia—the RPE-specific Lrp2 knockout mouse—VEGF expression is markedly reduced, leading to underdevelopment of the choriocapillaris and subsequent axial elongation ^(56)^. These findings highlight the retinal pigment epithelium as a central tissue coordinating vascular and structural remodeling under sustained myopic conditions.

**Figure 2.**
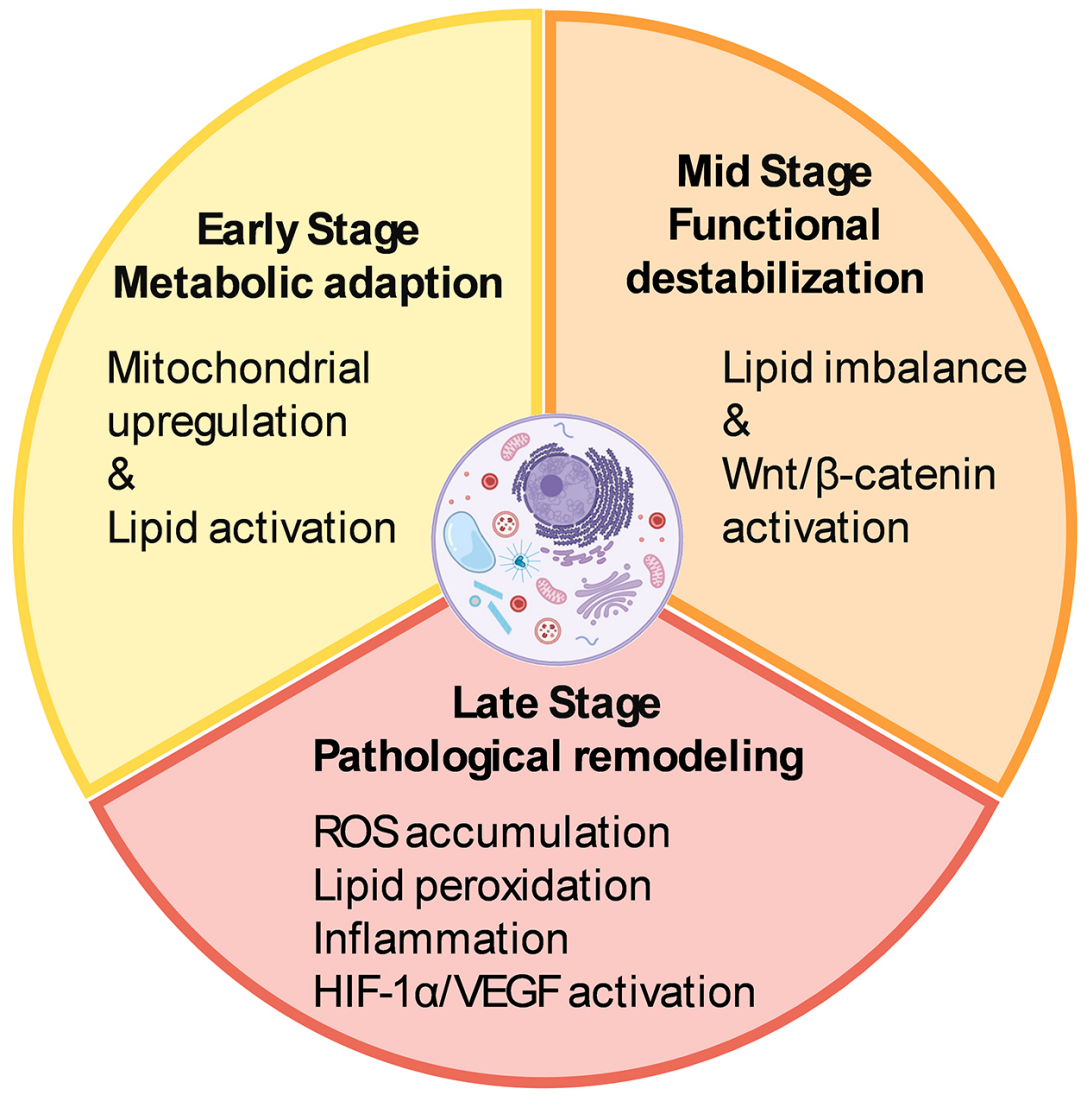
Temporal progression of metabolic remodeling in the myopic retina. The early stage features mitochondrial upregulation and lipid metabolism activation as adaptive responses. Mid-phase destabilization involves lipid imbalance and aberrant Wnt/β-catenin signaling. In the late stage, pathological remodeling is marked by oxidative stress, lipid peroxidation, chronic inflammation, and HIF-1α/VEGF pathway activation, creating a microenvironment prone to degeneration. The illustrations were created using BioRender (www.biorender.com). HIF-1α: hypoxia-inducible factor 1α; VEGF: vascular endothelial growth factor.

Persistent metabolic and molecular disturbances in the myopic retina create a microenvironment increasingly prone to dysfunction, even in the absence of overt pathological complications. If left unchecked, these changes may accelerate structural degradation and functional decline. Targeting specific metabolic vulnerabilities offers a promising route for intervention. Compounds such as crocetin ^(57)^, which mitigates oxidative stress, and fenofibrate ^(58)^, which modulates lipid metabolism, have shown encouraging protective effects on retinal integrity. Supporting the retina’s adaptive metabolic capacity may help reduce long-term vulnerability and slow retinal damage in progressive myopia. Beyond pharmacological approaches, violet light may also act as a modulator of retinal metabolism. Our laboratory has shown that violet light activates the transcription factor early growth response-1 (EGR1) ^(59)^ via the non-visual opsin OPN5 ^(60)^. As a pivotal early-response gene, EGR1 modulates inflammatory signaling across various biological contexts ^(61)^. This form of light-driven neuromodulation may represent a complementary strategy for influencing retinal metabolic responses during myopia progression. Figure 2 summarizes the stage-dependent metabolic remodeling and molecular alterations occurring in the myopic retina.

### Conclusions

The retina has emerged as a key regulator of myopia progression, extending beyond its traditional role in image formation. Recent findings indicate that disruptions in ON/OFF signaling, progressive remodeling of retinal layers, and stage-specific metabolic changes collectively contribute to the detection and transmission of defocus cues, ultimately promoting axial elongation. These alterations begin early, evolve gradually, and may culminate in irreversible retinal degeneration in long-standing high myopia. Future research should focus on elucidating stage-specific molecular and cellular events, mapping regional susceptibilities across retinal layers, and decoding the dynamic crosstalk between retinal, scleral, and choroidal compartments. A more comprehensive understanding of retinal mechanisms could not only advance our knowledge of myopia pathogenesis but also yield broader insights into neurodegenerative processes and support development of novel therapeutic strategies that enhance retinal resilience.

## Article Information

This article is based on the study, which received the Medical Research Encouragement Prize of The Japan Medical Association in 2024.

### Conflicts of Interest

None

### Acknowledgement

The authors thank all members of the Chorioretinal Biology Laboratory.

### Author Contributions

Conceptualization, literature review, figure design, and manuscript drafting: Yajing Yang and Yohei Tomita. Literature selection, thematic analysis, and critical revision: Deokho Lee and Toshihide Kurihara. Figure refinement and language editing: Kate Gettinger. Supervision and expert input: Kazuno Negishi, Kazuo Tsubota, Toshihide Kurihara, and Yohei Tomita. All authors reviewed and approved the final version of the manuscript.

### Commercial Relationships Disclosure

Yajing Yang, none; Yohei Tomita, none; Deokho Lee, none; Shin-ichi Ikeda, none; Xiaoyan Jiang, none; Kazuno Negishi, none; Kazuo Tsubota, Tsubota Laboratory, Inc.(E); Toshihide Kurihara, none.
